# Genomic landscape and clinical impact of *BRCA1/2* pathogenic variants in metastatic castration-resistant prostate cancer

**DOI:** 10.1038/s41698-026-01339-8

**Published:** 2026-02-26

**Authors:** Kazuki Iida, Fumihiko Urabe, Yuya Matsui, Kojiro Tashiro, Kentaro Yoshihara, Yusei Urabe, Takaaki Ishikawa, Juntaro Matsuzaki, Takahiro Kimura, Yoshimasa Saito

**Affiliations:** 1 Division of Pharmacotherapeutics, Keio University Faculty of Pharmacy, Tokyo, Japan; 2 Department of Urology, The Jikei University School of Medicine, Tokyo, Japan; 3 Division of Interdisciplinary Genetics and Nanomedicine, Research Center for Drug, Discovery, Keio University Faculty of Pharmacy, Tokyo, Japan; 4 Division of Gastroenterology and Hepatology, Department of Internal Medicine, Keio University School of Medicine, Tokyo, Japan

**Keywords:** Cancer, Genetics, Oncology

## Abstract

Poly (ADP-ribose) polymerase (PARP) inhibitors provide clinical benefit for patients with metastatic castration-resistant prostate cancer (mCRPC) harboring *BRCA1/2* pathogenic variants. This study aimed to investigate the genomic landscape of mCRPC and evaluate the prognostic and therapeutic impact of pathogenic *BRCA1/2* variants. We conducted a retrospective cohort study of 5893 patients with mCRPC registered in Japan. Overall survival (OS) was compared according to homologous recombination repair (HRR) gene alteration status and specific *BRCA1/2* pathogenic variants. Of 5893 patients, 2203 carried at least one pathogenic variant in HRR genes, and 792 had *BRCA1/2* pathogenic variants. Olaparib was recommended for all patients with *BRCA1/2* pathogenic variants, of whom 389 received the treatment. Patients with HRR gene alterations had significantly shorter OS than those without (*P* = 0.023). Among olaparib-treated patients, *BRCA1* pathogenic variants were significantly associated with worse OS compared with *BRCA2* pathogenic variants (*P* = 0.008). Within *BRCA2*-altered cases, *BRCA2* loss was associated with the most favorable OS (*P* < 0.001). Multivariate analysis confirmed *BRCA1* pathogenic variants as an independent adverse prognostic factor and *BRCA2* loss as an independent favorable factor in patients treated with olaparib. These findings highlight the clinical importance of detailed genomic annotation for precision oncology in mCRPC.

## Introduction

Prostate cancer is the second most common malignancy among men^1^. Although localized prostate cancer generally follows an indolent course, metastatic castration-resistant prostate cancer (mCRPC) is associated with a poor prognosis, despite a growing number of treatment options such as next-generation androgen receptor pathway inhibitors (e.g., abiraterone, enzalutamide, apalutamide) and taxane-based chemotherapy^2–5^. Emerging genomic studies show that mCRPC is heterogeneous^6,7^, and that clinical outcomes may vary based on underlying molecular alterations^8,9^. Among these, alterations in genes involved in androgen receptor signaling, cell cycle regulation, and DNA damage repair, particularly the homologous recombination repair (HRR) pathway, are of significant clinical relevance.

Targeted therapies guided by such molecular alterations have reshaped the therapeutic landscape. A prime example of genotype-matched therapy is olaparib, a poly (ADP-ribose) polymerase (PARP) inhibitor now approved for *BRCA*-altered breast, ovarian, pancreatic, and prostate cancers. The PROfound trial (NCT02987543) showed that olaparib improves radiographic progression-free survival (rPFS) and overall survival (OS) of patients with mCRPC harboring pathogenic *BRCA1*, *BRCA2*, or *ATM* variants significantly when compared with standard hormonal therapy^10^, with the effect being particularly significant in the *BRCA1* and *BRCA2* pathogenic variant groups^10^. Based on these findings, olaparib has been approved in Japan for patients with *BRCA1/2*-altered mCRPC.

Comprehensive genomic profiling (CGP), which enables tumor-agnostic molecular characterization, is increasingly integrated into clinical decision-making across oncology^11^. The National Cancer Center (NCC) is the leading implementer of precision oncology in Japan, and CGP testing became reimbursable in June 2019 following years of infrastructure development^12^. All genomic and clinical data generated through CGP testing are collected by the Center for Cancer Genomics and Advanced Therapeutics (C-CAT) with informed consent, creating a national-scale database for research and clinical monitoring^13^. This framework has the potential to facilitate population-scale analyses of pathogenic variants of prostate cancer. Against this background, we aimed to characterize the HRR pathway and *BRCA1/2* pathogenic variants and to evaluate the real-world treatment outcomes of patients with *BRCA1/2*-altered mCRPC who received olaparib.

## Results

### Study population

**Fig. 1:**
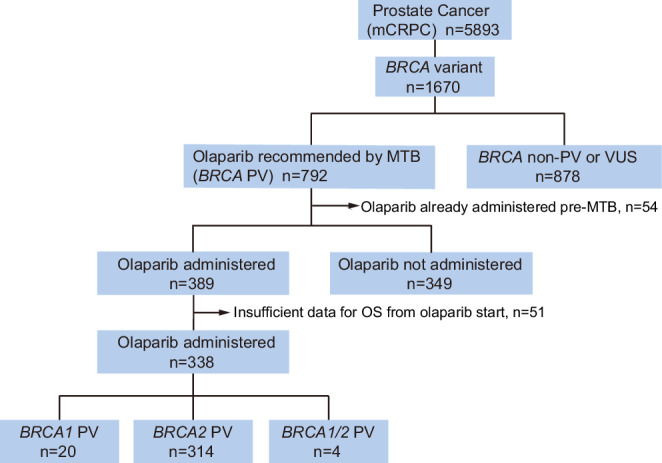
Flow diagram illustrating the selection of patients included in the study. A total of 5893 patients with prostate cancer were stratified according to *BRCA* alteration status and olaparib treatment. mCRPC metastatic Castration-Resistant Prostate Cancer, MTB Molecular Tumor Board, PV Pathogenic Variant, VUS Variant of Uncertain Significance.

Data from 5,893 patients with prostate cancer were collected from C-CAT. A detailed flow diagram depicting patient selection and categorization is shown in Fig. 1. Baseline clinical characteristics were defined at the time of specimen submission, based on information at the time of C-CAT registration. Analysis of the dataset revealed that patients with pathogenic *BRCA1/2* variants were more likely to have a family history suggestive of hereditary breast and ovarian cancer syndrome than the overall cohort (Supplementary Table 1). Patients with *BRCA* pathogenic variants were younger at the time of registration compared with those with wild-type *BRCA*. This result also indicates that, even among patients with prostate cancer, *BRCA1/2* alterations remain strongly associated with a positive family history (Supplementary Table 1). As shown in Supplementary Fig. 1A, the timing of Molecular Tumor Board (MTB) review in relation to systemic therapy revealed that the majority of patients (56.5%) underwent genomic testing during or after third-line treatment. This indicates that the MTB was convened primarily to guide treatment decisions beyond the third-line setting, particularly at or following the fourth-line. A gradual trend toward earlier MTB review was seen in recent cases (Supplementary Fig. 1B). The specimen types used for CGP are shown in Supplementary Fig. 1C**:** 12.6% surgical, and 30.5% archival biopsy at diagnosis, 11.8% rebiopsy, and 39.1% liquid biopsy. The distribution of specimen types across different CGP platforms is also presented in Supplementary Fig. 1C. In this study, the cohort evaluating the clinical outcomes of olaparib treatment consisted of patients who received olaparib after undergoing CGP testing. Accordingly, all patients initiated olaparib in the second-line setting or later. Although rare exceptions may occur, nearly all patients receiving olaparib after MTB review in our cohort had received prior treatments.

### Genomic landscape of prostate cancer patients from C-CAT

**Fig. 2:**
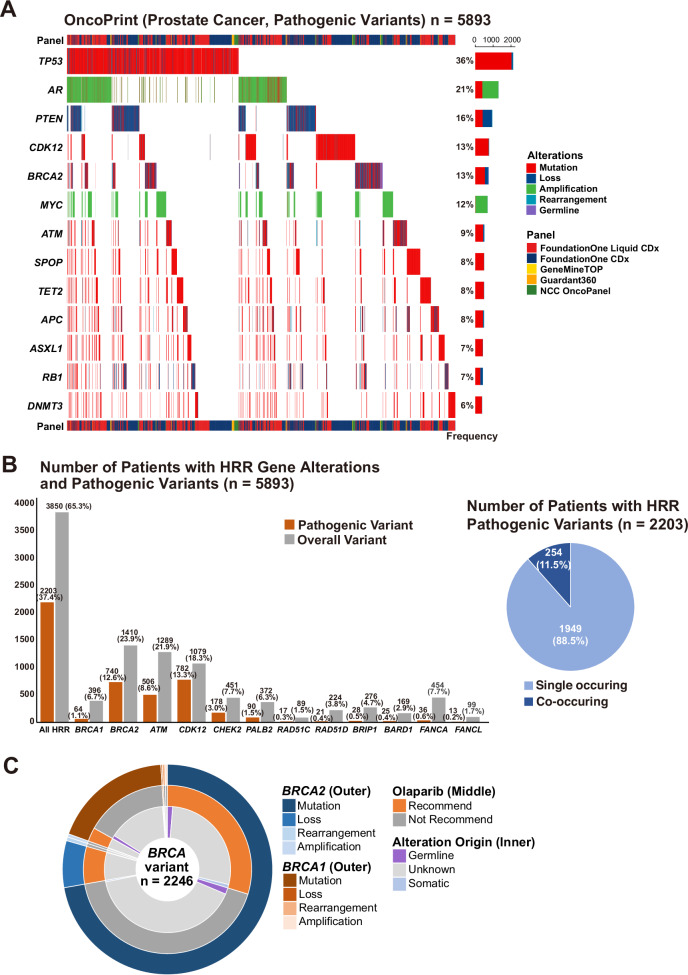
Genomic landscape and homologous recombination repair (HRR) gene alterations in prostate cancer. **A** Oncoprint illustrating the pathogenic variants in genes with a frequency of 5% or higher in prostate cancer (*n* = 5893). **B** Distribution of HRR gene alterations among 5893 patients. Bars indicate the number of patients with any detected variants and with pathogenic variants, excluding non-pathogenic variants and variants of uncertain significance (VUS). The pie chart (right) illustrates the proportion of patients with HRR pathogenic variants (*n* = 2203), showing single versus co-occurring variants. **C** Distribution of *BRCA* variant details (outer), olaparib recommendation (middle), and alteration origin (inner) (*n* = 2246).

The Oncoprint summarizes pathogenic variants excluding variants of uncertain significance (VUS) in genes with a frequency of 5% or higher in mCRPC (Fig. 2A). Most of the identified variants were either sequence-level mutations or large genomic rearrangements, such as gene deletions or amplifications. Pathogenic variants were most frequently observed in *TP53* (36%). *BRCA2*, *CDK12*, and *ATM* pathogenic variants were observed in 13%, 13%, and 9% of patients, respectively. As reported in the PROfound trial, we confirmed that *BRCA2* and *ATM*　pathogenic variants were confirmed to be mutually exclusive^10^.

A track indicating the CGP platform (FoundationOne CDx, FoundationOne Liquid CDx, GeneMineTOP, Guardant360, and NCC OncoPanel) was added in Fig. 2A. Most *DNMT3A* and *TET2* variants were identified through ctDNA-based assays (FoundationOne Liquid CDx and Guardant360), which may indicate a possibility of clonal hematopoiesis of indeterminate potential (CHIP) rather than tumor-derived alterations.

We additionally analyzed the AACR Project Genomics Evidence Neoplasia Information Exchange (GENIE) dataset (version 17.0) to characterize genomic alterations in Western patients with prostate cancer, and we incorporated these findings into an Oncoprint (Supplementary Fig. 2). In the C-CAT dataset used in the present study, the frequency of *CDK12* alterations was 13%, whereas it was 5% in the GENIE dataset. This indicates that *CDK12* alterations are substantially more frequent in the Japanese cohort compared with Western cohorts.

A focused analysis of 12 HRR-related genes, excluding VUS (Fig. 2B) showed that 2203 out of 5893 patients (37.4%) had ≥1 pathogenic variant (somatic or germline). Of these, 1,949 patients (88.5%) had a single HRR gene pathogenic variant, while 254 patients (11.5%) exhibited a co-occurring pathogenic variant (Fig. 2B). The most common co-alterations included *ATM* + *CHEK2*, *ATM* + *BRCA2*, *ATM* + *CDK12*, and *CDK12* + *CHEK2* (see Supplementary Table 2 for a complete list).

Figure 2C shows the overall distribution of *BRCA* variants in this cohort. A total of 2246 *BRCA* variants were identified among 5893 patients with mCRPC. Pathogenic variants of *BRCA1* or *BRCA2* (*BRCA1/2*) were detected in 792 patients (792/5893 = 13.4%), and 878 patients (878/5893 = 14.9%) carried non-pathogenic variants or VUS in *BRCA1/2* (Fig. 1). Among the 792 patients with pathogenic *BRCA1/2* variants, 895 distinct variants were identified: 65 *BRCA1* variants and 830 *BRCA2* variants (Supplementary Tables 3 and 4). Following MTB review, olaparib was recommended for 792 patients: 52 patients with *BRCA1* pathogenic variants, 728 patients with *BRCA2* pathogenic variants, and 12 patients with pathogenic variants in both *BRCA1* and *BRCA2*.

Among all cases included in this analysis, 326 patients were submitted for genomic profiling using the NCC OncoPanel, along with 49 analyzed using the GenMineTOP assay, both of which allow distinction between somatic and germline variants. Among these, the NCC OncoPanel identified germline *BRCA1/2* pathogenic variants in 40 patients (12.3%), and GenMineTOP identified these variants in eight patients (16.3%). Specifically, 29 patients (7.7%) harbored pathogenic germline *BRCA2* variants, and two (0.5%) harbored pathogenic germline *BRCA1* variants. Notably, the most common founder mutation in *BRCA1* was L63*, while *BRCA2* most commonly harbored the R2318* and I1859fs*3 variants (Supplementary Tables 3 and 4). Overall, 12 patients had concurrent variants of both *BRCA1* and *BRCA2*; the detailed variant profiles for these cases are shown in Supplementary Table 5.

### Oncological outcomes in patients with prostate cancer

**Fig. 3:**
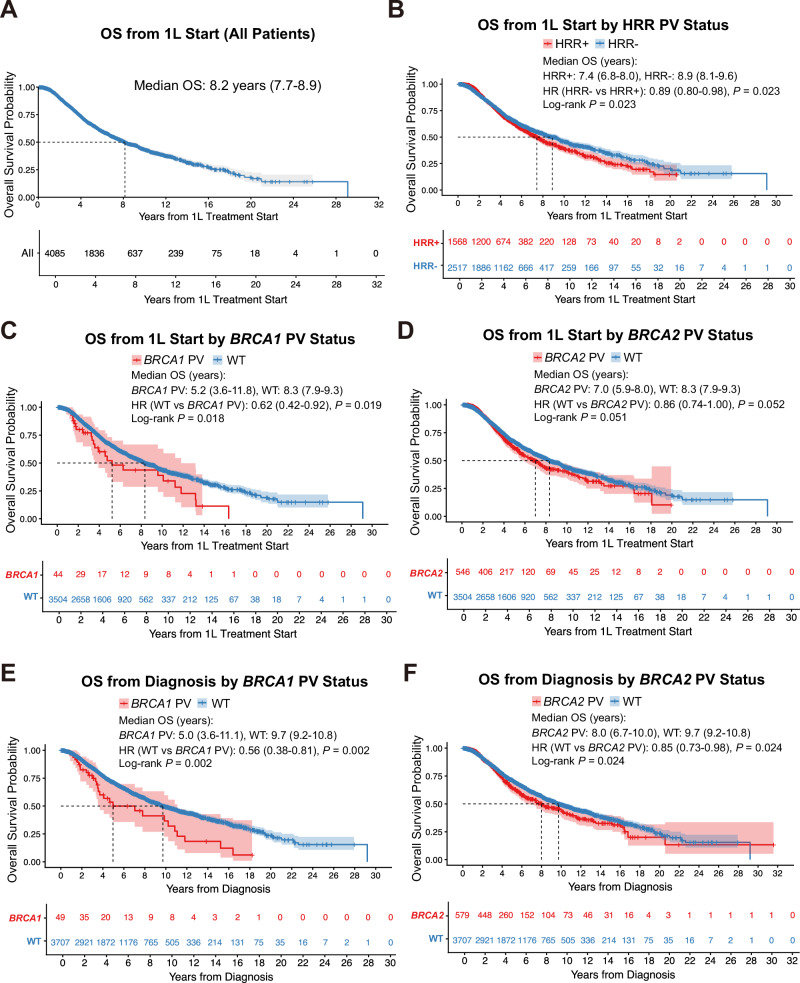
Survival analysis in prostate cancer patients with homologous recombination repair (HRR) gene variants. **A** Kaplan–Meier curves for overall survival (OS) from the start of first-line (1 L) treatment in patients eligible for prognostic analysis. **B** Kaplan–Meier curves for OS from the start of 1 L treatment in patients with or without pathogenic HRR gene variants. **C** Kaplan–Meier curves for OS from the start of 1 L treatment in patients with *BRCA1* pathogenic variants (PVs) compared with wild-type (WT). **D** Kaplan–Meier curves for OS from the start of 1 L treatment in patients with *BRCA2* PVs compared with WT. **E** Kaplan–Meier curves for OS from the date of prostate cancer diagnosis in patients with *BRCA1* PVs compared with WT. **F** Kaplan–Meier curves for OS from the date of prostate cancer diagnosis in patients with *BRCA2* PVs compared with WT.

A total of 5893 patients with prostate cancer were submitted for CGP; however, due to insufficient data regarding 1st line systemic therapy, only 4085 of these were ultimately included in the analysis (44 with *BRCA1*, 546 with *BRCA2*, and 9 with both *BRCA1* and *BRCA2* pathogenic variants: Fig. 3A–D). OS from the date of 1st line systemic therapy (OS_1^st^_line) is illustrated in Fig. 3A, with a median OS of 8.2 years (95%CI, 7.7–8.9). Next, we compared outcomes according to pathogenic HRR variant status, excluding non-pathogenic variants or VUS. Patients harboring HRR pathogenic variants exhibited significantly shorter OS (OS_1^st^_line) than those without such variants (log-rank *P* = 0.023; Fig. 3B). With respect to metastatic sites, among the 4085 patients for whom OS_1st_line was evaluable, prognosis differed according to metastatic pattern: patients with brain metastases had the worst outcomes, followed by those with liver excluding brain metastases, whereas patients with lung metastases (excluding liver and brain metastases) and bone-only metastases (excluding brain, liver, and lung metastases) demonstrated relatively more favorable survival (Supplementary Fig. 3A). Importantly, this trend was consistently observed in both patients with and without pathogenic HRR variants (Supplementary Fig. 3B, 3C).

Furthermore, patients with a pathogenic *BRCA1* variant had significantly shorter OS_1^st^_line than those without a pathogenic *BRCA1*/*BRCA2* variant (wild-type [WT]) (log-rank *P* = 0.018; Fig. 3C). A similar trend was observed for patients with a pathogenic *BRCA2* variant (log-rank *P* = 0.051; Fig. 3D). These associations remained significant when OS was calculated from the date of prostate cancer diagnosis (log-rank *P* = 0.002 and *P* = 0.024, respectively; Fig. 3E, 3F). In this analysis, 4,325 patients were included, comprising 49 with *BRCA1*, 579 with *BRCA2*, and 10 with both *BRCA1* and *BRCA2* pathogenic variants.

We also compared OS between HRR-positive but *BRCA*-negative (HRR + /*BRCA*–) patients and HRR-wild-type (HRR–) patients. Although no statistically significant difference was observed (*P* = 0.222), the HRR + /*BRCA*– group showed a numerically shorter OS than the HRR– group, indicating a trend toward poorer prognosis. This observation is consistent with trends reported in a previous study^14^ (Supplementary Fig. 4A).

As shown in Fig. 1, among the 792 patients for whom olaparib was recommended, 389 (49.1%) ultimately received the therapy. In the olaparib-treated cohort, patients harboring *BRCA1/2* pathogenic variants had a median OS of 17.4 months (95% CI, 15.1–20.9) following treatment initiation. We compared the OS between *BRCA1/2* pathogenic variant–positive patients who received olaparib and those who were recommended olaparib by the MTB but ultimately did not receive treatment. Although the olaparib-treated group showed a tendency toward better survival, the difference was not statistically significant (Supplementary Fig. 4B). Regarding patients who did not receive olaparib treatment, we speculate that this may reflect a mixture of two opposing clinical scenarios: patients who were recommended olaparib but experienced rapid disease progression before treatment could be initiated, and those who were recommended olaparib but remained well controlled on their current therapy and therefore did not require a treatment change.

**Fig. 4:**
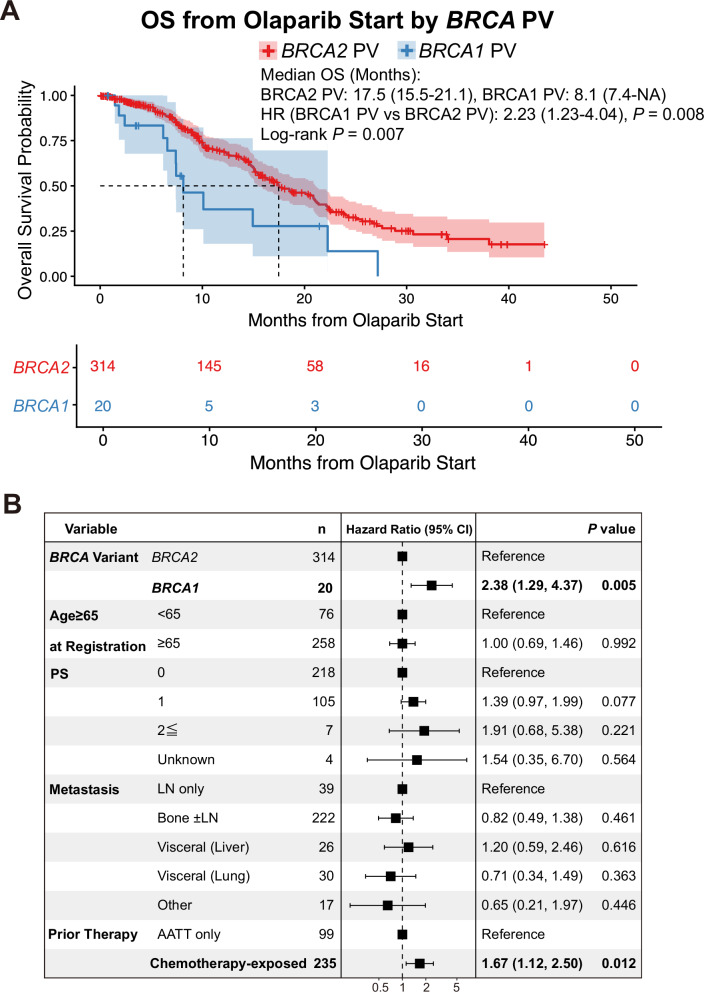
Survival analysis of prostate cancer patients with *BRCA1* or *BRCA2* pathogenic variants (PVs) treated with olaparib. **A** Kaplan–Meier curves for overall survival (OS) from the start of olaparib treatment, stratified by pathogenic *BRCA* variant status (*BRCA1* or *BRCA2* pathogenic variants). **B** Forest plot showing hazard ratios (HRs) and 95% confidence intervals (CIs) from multivariate analysis of OS from the start of olaparib treatment, stratified by pathogenic *BRCA* variant status (*BRCA1* or *BRCA2* pathogenic variants). Metastatic burden was classified into four categories: lymph-node (LN)–only metastasis; bone metastasis with or without lymph-node involvement; visceral metastasis (lung or liver); and “other,” which included all remaining metastatic sites. Prior therapy before olaparib initiation was classified into two categories: androgen-axis–targeted therapy (AATT) alone, and chemotherapy-exposed (including taxane-based chemotherapy and platinum-based therapy).

Next, we compared OS following olaparib initiation (OS_olaparib) between patients harboring *BRCA1* and *BRCA2* pathogenic variants. In this cohort, only two patients received concomitant abiraterone and olaparib, and no patients received other next-generation androgen receptor pathway inhibitors or chemotherapy concurrently with olaparib. The median OS_olaparib was 17.5 months for *BRCA2* pathogenic variants and 8.1 months for *BRCA1* pathogenic variants. OS_olaparib was significantly shorter in patients with *BRCA1* pathogenic variants than in those with *BRCA2* variants (unadjusted HR: 2.23 [95% CI, 1.23–4.04], *P* = 0.008; Fig. 4A). Notably, there were no major differences in baseline characteristics between *BRCA1* and *BRCA2* groups (Supplementary Table 6).

We further evaluated OS_olaparib according to treatment exposure prior to olaparib initiation. Compared with patients who had received androgen-axis–targeted therapy (AATT) alone, those previously treated with taxane-based chemotherapy exhibited significantly shorter OS_olaparib (unadjusted HR: 0.56 [95% CI, 0.38–0.82], *P* = 0.003; Supplementary Fig. 5). In contrast, no statistically significant difference in OS_olaparib was observed among patients who had received platinum-based therapy, potentially reflecting the limited sample size of this subgroup (unadjusted HR: 0.71 [95% CI, 0.35–1.45], *P* = 0.352; Supplementary Fig. 5).

In addition, 27 patients (27/314; 8.6%) had been diagnosed with neuroendocrine prostate cancer (NEPC) based on pathological or clinical criteria prior to olaparib initiation; notably, all 27 patients harbored pathogenic *BRCA2* mutations. OS_olaparib did not differ significantly between patients with and without a diagnosis of NEPC (unadjusted HR: 1.31 [95% CI, 0.72–2.38], *P* = 0.379; Supplementary Fig. 6).

The distribution of metastatic sites in the olaparib cohort was as follows: brain metastases, 2/338 (0.6%); liver metastases (excluding brain metastases), 26/338 (7.7%); lung metastases (excluding brain and liver metastases), 30/338 (8.9%); and bone metastases (excluding brain and visceral metastases), 225/338 (66.6%). Because only two patients had brain metastases, outcome analyses were conducted after excluding these cases; no significant differences in OS_olaparib according to metastatic site were observed (Supplementary Fig. 7).

Multivariate analysis confirmed that *BRCA1* pathogenic variant and prior exposure to chemotherapy were independently associated with a worse prognosis (adjusted HR: 2.38 [95% CI, 1.29–4.37], *P* = 0.005 and adjusted HR: 1.67 [95% CI, 1.12–2.50], *P* = 0.012, respectively; Fig. 4B). Because the group of concurrent *BRCA1* + *2* pathogenic variants consisted of only four cases, Kaplan–Meier and multivariable analyses were not performed. Nevertheless, given the clinical rarity and potential importance of this subgroup, we report that the median OS_olaparib was 11.2 months, and patient characteristics are provided in Supplementary Table 6.

**Fig. 5:**
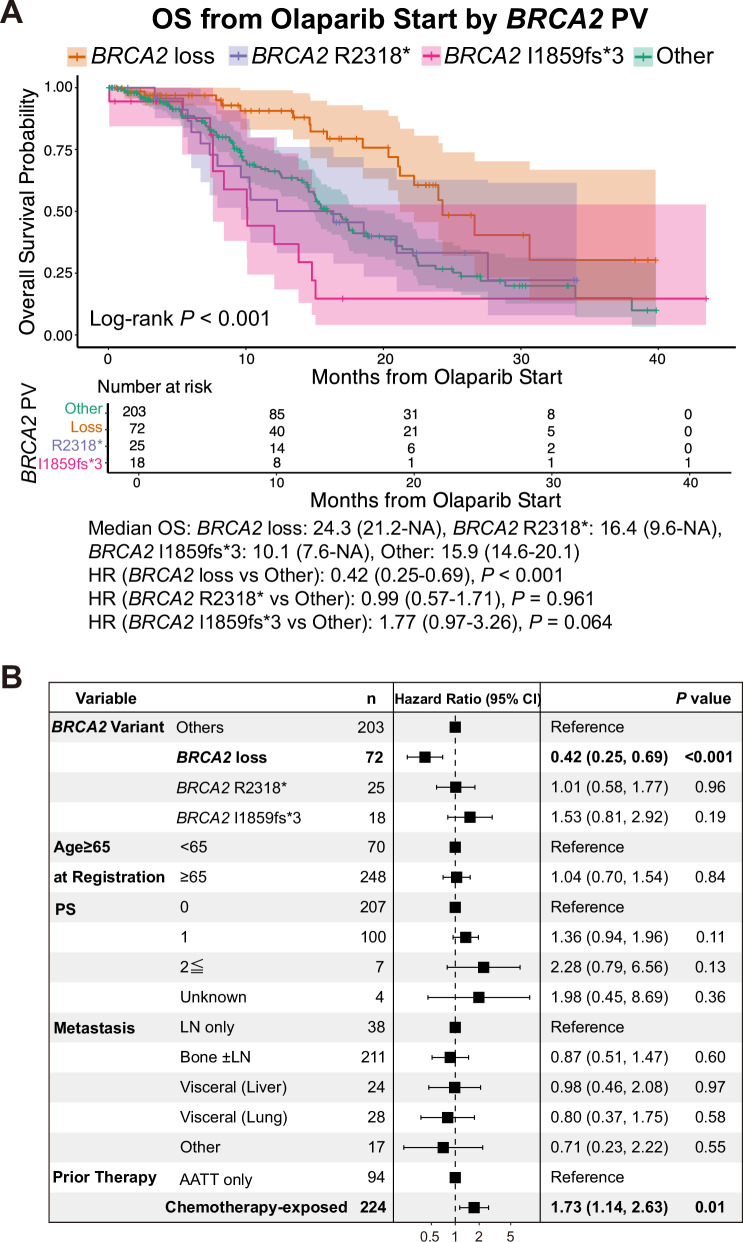
Survival analysis of prostate cancer patients with *BRCA2* pathogenic variants (PVs) treated with olaparib. **A** Kaplan–Meier curves for OS from the start of olaparib treatment, stratified by pathogenic *BRCA2* variant status (*BRCA2* loss, R2318*, I1859fs*3, or others). **B** Forest plot showing HRs and 95% CIs from multivariate analysis of OS from the start of olaparib treatment, stratified by pathogenic *BRCA2* variant status (*BRCA2* loss, R2318*, I1859fs*3, or others). Metastatic burden was classified into four categories: lymph-node (LN)–only metastasis; bone metastasis with or without lymph-node involvement; visceral metastasis (lung or liver); and “other,” which included all remaining metastatic sites. Prior therapy before olaparib initiation was classified into two categories: androgen-axis–targeted therapy (AATT) alone, and chemotherapy-exposed (including taxane-based chemotherapy and platinum-based therapy).

Furthermore, we investigated OS_olaparib according to the most frequent *BRCA2* variant types among patients with *BRCA2* pathogenic variants: loss, R2318* nonsense variant, I1859fs*3 frameshift variant, and a group comprising all other variants. Differences in characteristics among these subgroups are shown in Supplementary Table 7. Patients with loss of *BRCA2* had a more favorable prognosis than those in the other groups (unadjusted HR: 0.42 [95% CI, 0.25–0.69], *P* < 0.001; Fig. 5A), while those with the I1859fs*3 variant showed the poorest survival trend, although the data were not statistically significant (unadjusted HR: 1.77 [95% CI, 0.97–3.26], *P* = 0.064; Fig. 5A). Multivariate analysis also identified loss of *BRCA2* as an independent predictor of a favorable prognosis (adjusted HR: 0.42 [95% CI, 0.25–0.69], *P* < 0.001; Fig. 5B).

## Discussion

In this nationwide genomic analysis of more than 5000 patients with mCRPC, we outline the genomic landscape and assess the clinical relevance of *BRCA1/2* alterations, which are presently the approved molecular target for PARP inhibitor therapy in prostate cancer. To the best of our knowledge, this study represents the largest and most comprehensive real-world genomic dataset derived from patients with prostate cancer to date. Moreover, this is the first study to evaluate the real-world efficacy of olaparib in patients with mCRPC according to *BRCA* pathogenic variant status.

Data regarding the prevalence of *BRCA1/2* pathogenic variants in Japanese populations remains limited. A previous study by Momozawa et al. reported the frequency of germline pathogenic variants in a mixed-stage prostate cancer cohort, with *BRCA1*, *BRCA2*, and *ATM* pathogenic variants detected in 0.2%, 1.1%, and 0.5% of cases, respectively^15^. The relatively low prevalence was likely attributable to the exclusion of somatic pathogenic variants, as well as the predominance of early-stage disease, in that cohort. Notably, the same study identified R2318* and I1859fs3 as recurrent germline *BRCA2* variants, findings corroborated by our analysis, in which these two alterations were among the most common *BRCA2* variants. As our dataset is derived from the FoundationOne CDx and Liquid CDx assays, which do not distinguish somatic from germline variants, it remains unclear whether these alterations are of somatic or germline origin; however, recurrence of R2318* in *BRCA2* across multiple tumor types as a germline variant, including colorectal cancer in Japanese patients^16^, suggests that this variant may represent a population-specific pathogenic germline founder mutation.

In our cohort, pathogenic variants in HRR pathway genes were identified in 37.4% of patients, with *BRCA1/2* pathogenic variants accounting for 13.4%, a prevalence largely consistent with reports from Western populations^6,9,17^. Furthermore, our findings closely align with those of the Japanese ZENSHIN study, which analyzed HRR pathogenic variants using archival tumor specimens and reported a *BRCA1/2* pathogenic variant prevalence of 13.3%^18^, reinforcing the generalizability of our results within Japanese clinical settings. Interestingly, *CDK12* pathogenic variants were observed in 13% of our patients (Fig. 2B), whereas *CDK12* alterations were detected at a frequency of approximately 5% in the Western cohort from the GENIE dataset (Supplementary Fig. 2). This frequency in the Japanese cohort was therefore substantially higher than the 5–7% prevalence reported in Western populations^19^.

*CDK12* biallelic inactivation is associated with a distinctive genomic instability signature involving focal tandem duplications and elevated gene fusion rates^20,21^. Ethnic variation may explain these findings, at least partly. A higher prevalence of *CDK12* pathogenic variants was reported in East Asian patients with mCRPC (12.9%) than in non-East Asians (4.2%), supporting a potential ancestry-associated disparity in pathogenic variant distribution^18^. Our findings align with the ZENSHIN study, which reported a similar *CDK12* pathogenic variant rate of 13.3% in a Japanese cohort^18^.

While the prognostic impact of *BRCA* pathogenic variants in mCRPC is inconsistent across studies^19,22–25^, *BRCA* alterations are generally associated with aggressive clinical features and unfavorable survival outcomes^26–28^. The CAPTURE study, a large multicenter European cohort of 729 mCRPC patients, evaluated treatment patterns and outcomes according to somatic and germline HRR alterations, demonstrating significantly poorer survival in patients with *BRCA1/2* pathogenic variants^14^. Consistent with these observations, our analysis of the C-CAT cohort revealed that patients harboring HRR pathway pathogenic variants have a significantly shorter OS than those without such alterations. The PROfound trial demonstrated that olaparib confers a significant benefit in terms of rPFS and OS among patients with *BRCA1/2* or *ATM* pathogenic variants, with an exploratory subgroup analysis reporting a 78% reduction in progression risk in *BRCA1/2*-altered patients^10^. Consistent with these findings, we found that patients with *BRCA1/2* pathogenic variants in our real-world cohort had a median OS of 17.4 months (95% CI: 15.1–20.9) following olaparib initiation, aligning closely with outcomes reported in the PROfound trial^10^. Importantly, prior translational analyses suggested that *BRCA1* pathogenic variants may show a poorer response to olaparib than *BRCA2* pathogenic variants^10,29^. Our data support this observation, as patients with *BRCA1* pathogenic variants alone exhibited significantly shorter OS, potentially reflecting limited therapeutic efficacy. These findings highlight the need for further studies stratifying clinical outcomes according to specific *BRCA* genotypes and mutation zygosity.

*BRCA1/2* alterations are the only currently approved molecular targets for PARP inhibitor therapy in Japan for prostate cancer. While it is conceivable that the therapeutic efficacy of PARP inhibitors may differ depending on the specific *BRCA* pathogenic variant, no prior studies have systematically evaluated whether clinical outcomes vary by individual variant. To the best of our knowledge, this is the first study to investigate olaparib efficacy in relation to distinct *BRCA* pathogenic variants in a real-world setting. We found that patients harboring *BRCA* loss exhibited longer OS following olaparib treatment than those with other types of *BRCA* pathogenic variants. Notably, *BRCA2* loss is less susceptible to reversion mutations, a major mechanism of PARP inhibitor resistance^30^. Moreover, recent case reports have described two patients with *BRCA2* loss who also harbored concurrent *RB1* alterations (splice-site variants or copy-number loss) and experienced marked and durable responses to PARP inhibitor therapy. Although *BRCA2* loss is generally expected to retain PARP inhibitor sensitivity due to a lower likelihood of reversion events, these cases further suggest that co-occurring genomic alterations—such as *RB1* abnormalities—may additionally modulate therapeutic response. Collectively, these observations support the emerging concept that the efficacy of PARP inhibitors may vary according to the specific pattern of *BRCA* aberrations^31^. In our current analysis, we also observed that patients with the I1859fs*3 variant showed a trend toward poorer survival, although this did not reach statistical significance. These findings suggest that, with larger datasets and extended follow-up, it may become possible to predict the efficacy of PARP inhibitors more precisely based on individual *BRCA* variant status.

In our cohort, prior exposure to taxane-based chemotherapy was associated with shorter OS following the initiation of olaparib. Notably, post hoc subgroup analyses of the PROfound trial stratified by prior taxane use among patients with alterations in only *BRCA1* or *BRCA2* demonstrated that olaparib retained a degree of clinical benefit in both taxane-naïve and post-taxane settings, although an effect of prior taxane exposure on OS was observed^29^. However, these subgroup findings should be interpreted with caution, as they are exploratory in nature and not powered for definitive comparisons. Moreover, the observed association likely reflects more advanced disease status or a higher disease burden at the time of olaparib initiation in patients previously treated with taxane, rather than a reduced intrinsic efficacy of olaparib itself. Therefore, the optimal sequencing of PARP inhibitors and taxane-based chemotherapy in mCRPC remains unclear and warrants further investigation in prospective, adequately powered studies.

This study has several limitations. (1) It employed a retrospective cohort design, which may introduce selection bias, and some cases were excluded because of incomplete clinical or prognostic data, which may have impacted the generalizability of the findings. (2) Standardized imaging and clinical evaluation data were not uniformly available, preventing reliable assessment of progression-free survival and cancer-specific survival. (3) The available CGP data did not allow us to determine whether *BRCA1/2* alterations were of somatic or germline origin in all cases or to distinguish homozygous from hemizygous deletions. (4) The database lacked Information on prior local treatments, such as surgery or radiation therapy, which may have influenced survival outcomes. (5) In Japan, a certain number of patients have been diagnosed with *BRCA* pathogenic variants using BRACAnalysis rather than CGP and may receive olaparib earlier in the disease course. Although this could limit the generalizability of our findings to the broader population, the increasing use of CGP in recent years supports the clinical relevance of our analysis.

In conclusion, we conducted the largest genomic analysis to date of advanced prostate cancer in a Japanese population. The overall distribution of frequently altered genes was largely consistent with previous reports. Notably, R2318* and I1859fs*3 emerged as recurrent *BRCA2* alterations, supporting their potential role as germline founder mutations specific to the Japanese population. Among patients treated with olaparib, those harboring *BRCA1* pathogenic variants had inferior outcomes to those with *BRCA2* pathogenic variants, suggesting differential therapeutic responsiveness to PARP inhibitors. Moreover, within the *BRCA2* variant subgroup, patients with *BRCA2* loss had the most favorable response to olaparib, highlighting the potential importance of pathogenic variant subtype in predicting treatment efficacy. These findings underscore the clinical relevance of detailed genomic profiling to inform precision treatment strategies in prostate cancer.

## Methods

### Study design and patients

Genomic and clinical data from 5,893 patients with prostate cancer were obtained from the C-CAT database on June 23, 2025. Five CGP tests have been approved and are reimbursed under the national health insurance system in Japan: FoundationOne CDx (F1CDx; tumor tissue), FoundationOne Liquid CDx and Guardant360 (plasma cfDNA), and paired tumor–normal OncoGuide NCC OncoPanel System and GeneMineTOP^13,32^. Test selection is generally at the physician’s discretion, with liquid biopsy used mainly when tumor tissue is unavailable or inadequate.

The study was reviewed and approved by the Institutional Review Board of Keio University (approval number: 251001-1) and by the C-CAT Data Utilization Review Board (CDU2023-039). All procedures were conducted in accordance with the ethical standards of the Declaration of Helsinki. Data were extracted through the C-CAT Research-Use Portal, with all patients providing written informed consent for the secondary use of their clinical information and CGP testing results for research purpose.

### Variant analysis

Tumor gene profiling was performed by next-generation sequencing using one of the five approved CGP tests. The results of tumor genomic profiling are discussed by an MTB comprising oncologists, geneticists, and other specialists^33^. In this study, based on the Clinical Knowledge Database (CKDB), HRR gene alterations were defined as pathogenic variants if their clinical significance was classified as “Pathogenic”, “Likely Pathogenic”, “Oncogenic”, or “Likely Oncogenic”. The 12 predefined HRR-related genes included *BRCA1, BRCA2, ATM, CDK12, CHEK2, PALB2, RAD51C, RAD51D, BRIP1, BARD1, FANCA*, and *FANCL*. Patients were considered alteration-positive if any of these classifications were detected in any of the 12 genes. Conversely, patients with no detectable pathogenic variants, or those with VUS, were categorized as “negative variant”. This category also included those with alterations annotated as “Inconclusive”, “Benign”, “Likely Benign”, or “Not Provided”, as these were not considered to have clear pathogenic or oncogenic relevance. Patients registered in C-CAT are encouraged to have their clinical data updated continuously, even after MTB review of tumor gene profiling. This longitudinal data input enables tracking of treatment selection and clinical outcomes in relation to the presence or absence of pathogenic variants.

### Study outcomes

The primary outcome was OS, stratified according to HRR gene alteration status and olaparib treatment. OS_1^st^_line was defined as the time from initiation of first-line systemic therapy to death from any cause or last follow-up. This outcome was analyzed according to the presence or absence of pathogenic alterations in HRR-related genes. OS_olaparib was defined as the time from the initiation of olaparib among patients with *BRCA1/2* pathogenic variants to the date of death or last follow-up. Patients alive at the data cut-off point were censored at the date of last contact.

### Statistical analysis

Patient characteristics are summarized as the median (IQR) for continuous variables, and as percentages (%) for categorical variables. Between-group comparisons were conducted using the Wilcoxon rank sum test or Kruskal–Wallis rank sum test (continuous variables) and Fisher’s exact test or Pearson’s chi-squared test (categorical variables). Kaplan–Meier survival curves were generated for OS_1^st^_line and OS_olaparib, with subgroup differences assessed by the log-rank test. Multivariable analysis for OS_olaparib was performed using a Cox proportional hazards model, incorporating age at registration, performance status (PS) at registration, sites of metastases, and prior treatment histories as covariates. These variables were selected a priori, based on prior literature, as standard pre-treatment prognostic factors that could be reliably ascertained from our database^34^. A two-sided *P* value < 0.05 was considered significant. All statistical analyses were performed using R software version 4.5.0 (R Foundation for Statistical Computing, Vienna, Austria).

## Supplementary information

Supplementary Materials

## Data Availability

The data analyzed in this study were obtained from the C-CAT under a data use agreement. These data are not publicly available; access requires submission of a formal application and approval by the C-CAT Data Utilization Review Board.
