# Supplementary material for: Genomic landscape and clinical impact of BRCA1/2 pathogenic variants in metastatic castration-resistant prostate cancer
Source: NPJ Precis Oncol. 2026 Feb 26;10:145. doi: 10.1038/s41698-026-01339-8 (PMC13061945; doi:10.1038/s41698-026-01339-8)
Supplement: Supplementary file 1 — Supplementary Materials [file 41698_2026_1339_MOESM1_ESM.pdf]

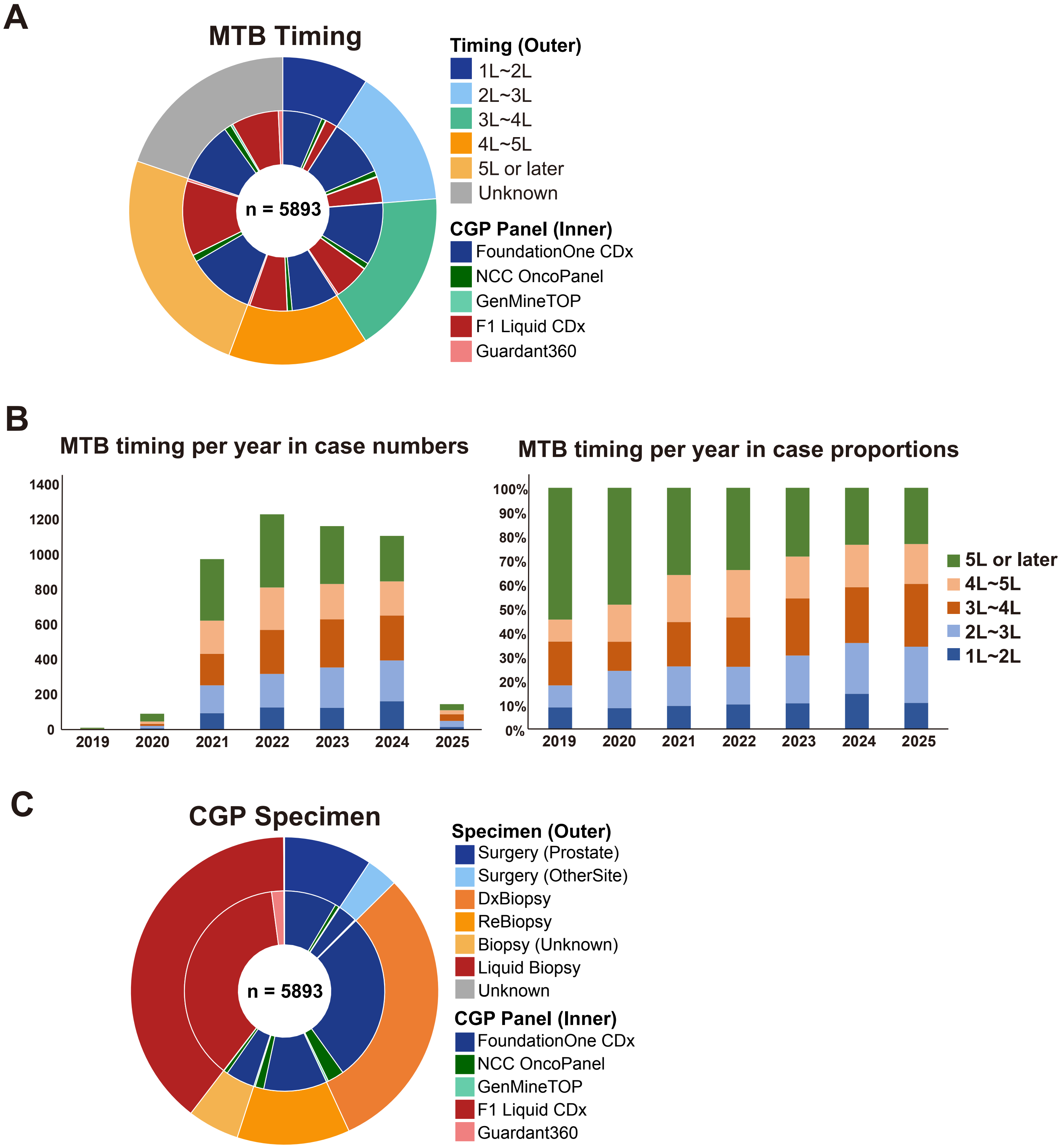


**Supplementary Figure 1.**
**Molecular tumor board (MTB) timing and comprehensive genomic profiling (CGP) specimen in patients with prostate cancer.**
**A)** Timing of MTB reviews (outer) and types of CGP panel assays (inner) performed in the overall cohort (n=5,893).
**B)** Annual trends in MTB timing.
(Left) MTB timing per year in case numbers. (Right) MTB timing per year in case proportions.
**C)** Types of specimens for CGP (outer) and CGP panel assays (inner) in the overall cohort (n=5,893). “Rebiopsy” is defined as samples obtained after first-line systemic therapy.

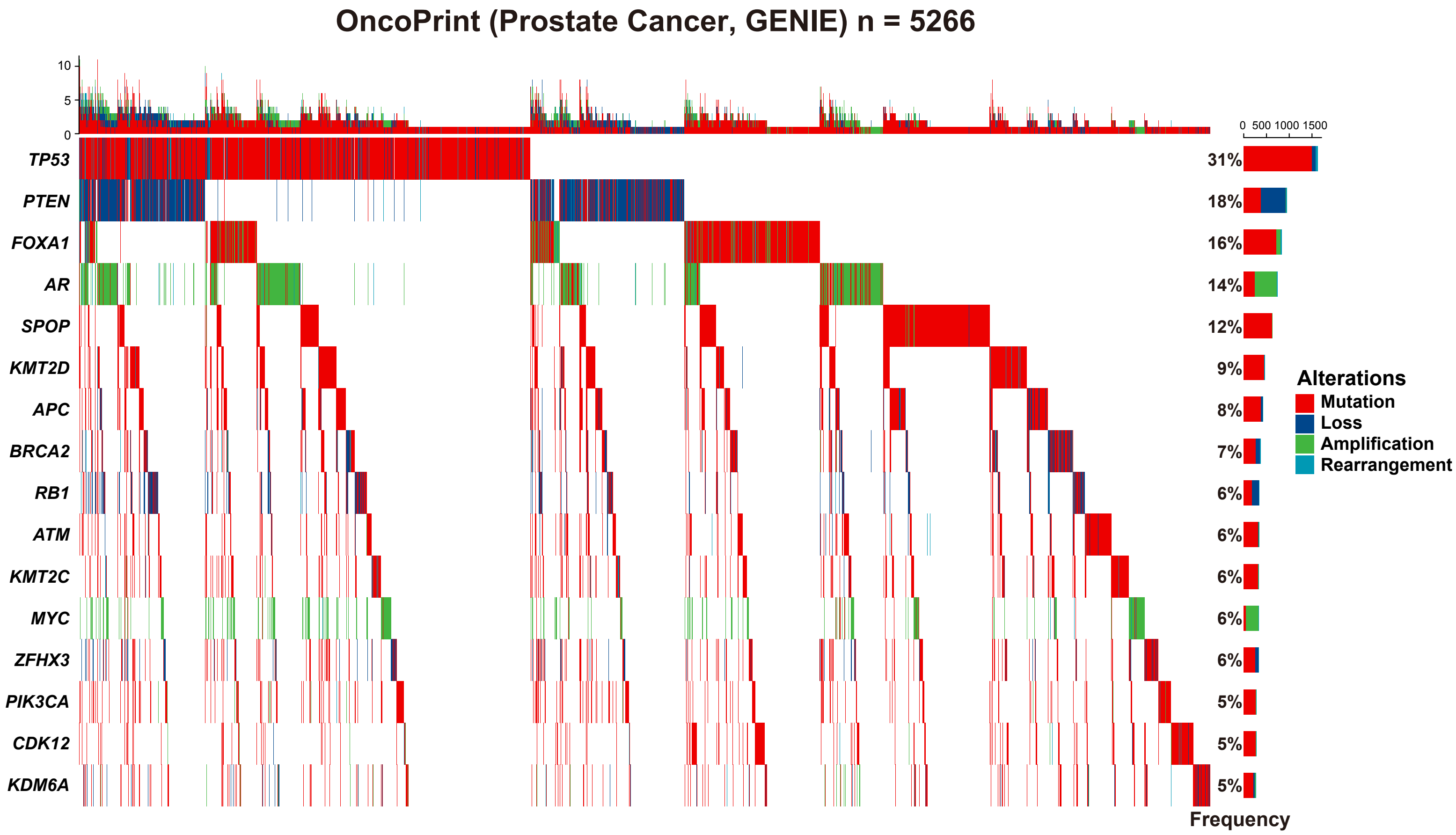


**Supplementary Figure 2.**

Oncoprint summarizing the distribution of gene variants in the AACR Project Genomics Evidence Neoplasia Information Exchange (GENIE) dataset (version 17.0) to investigate genomic alterations in Western patients with prostate cancer (n=5,266).

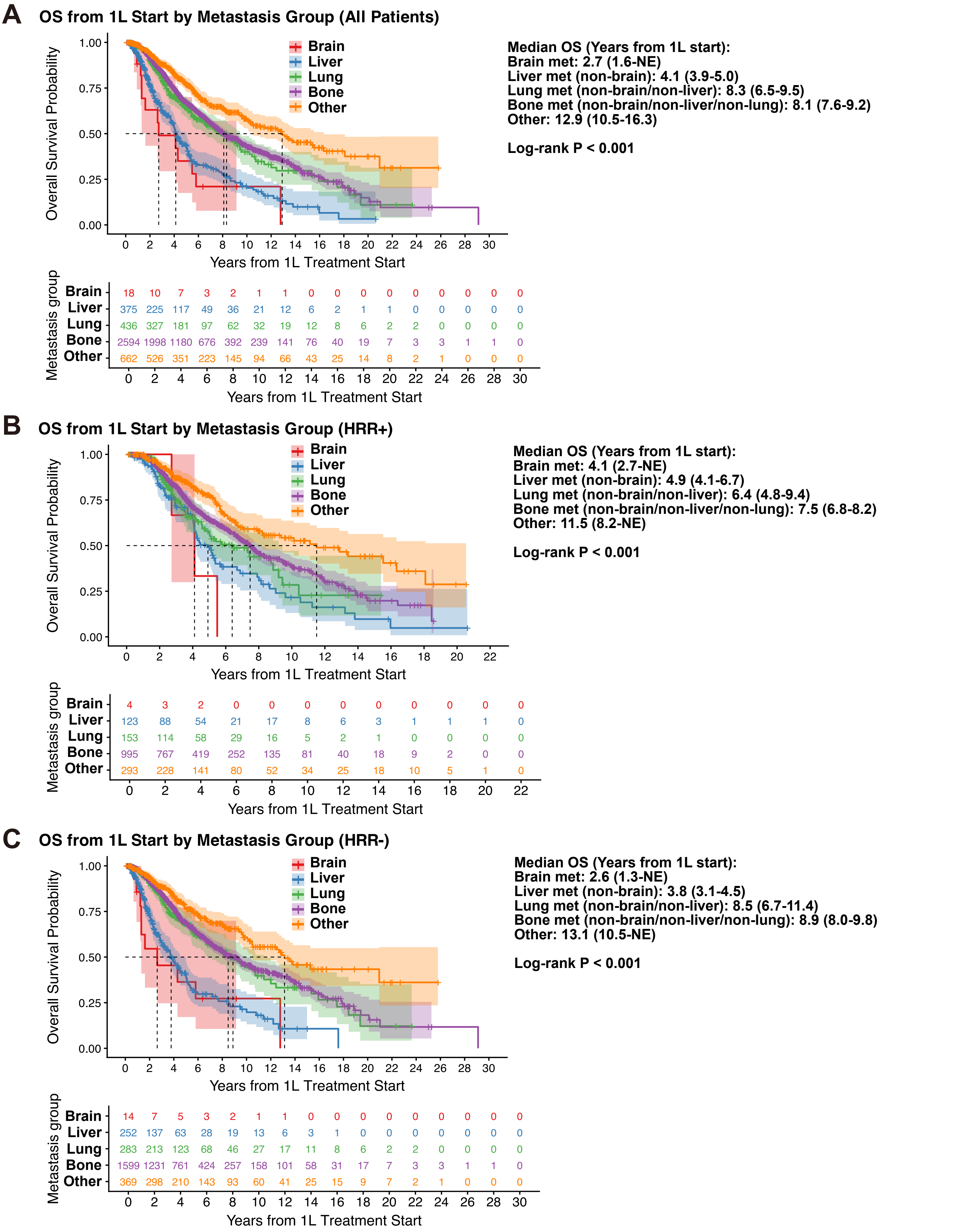


**Supplementary Figure 3.**

**Survival analyses stratified by metastatic site in prostate cancer patients.**

**A)** Kaplan–Meier curves for overall survival (OS) from the initiation of first-line (1L) treatment, stratified by metastatic site (brain, liver, lung, bone, and other sites).

**B)** Kaplan–Meier curves for OS from the initiation of 1L treatment among patients with pathogenic HRR gene variants, stratified by metastatic site (brain, liver, lung, bone, and other sites).

**C)** Kaplan–Meier curves for OS from the initiation of 1L treatment among patients without pathogenic HRR gene variants, stratified by metastatic site (brain, liver, lung, bone, and other sites).

Metastatic site definitions: liver metastases (excluding brain metastases), lung metastases (excluding brain and liver metastases), and bone metastases (excluding brain, liver, and lung metastases).

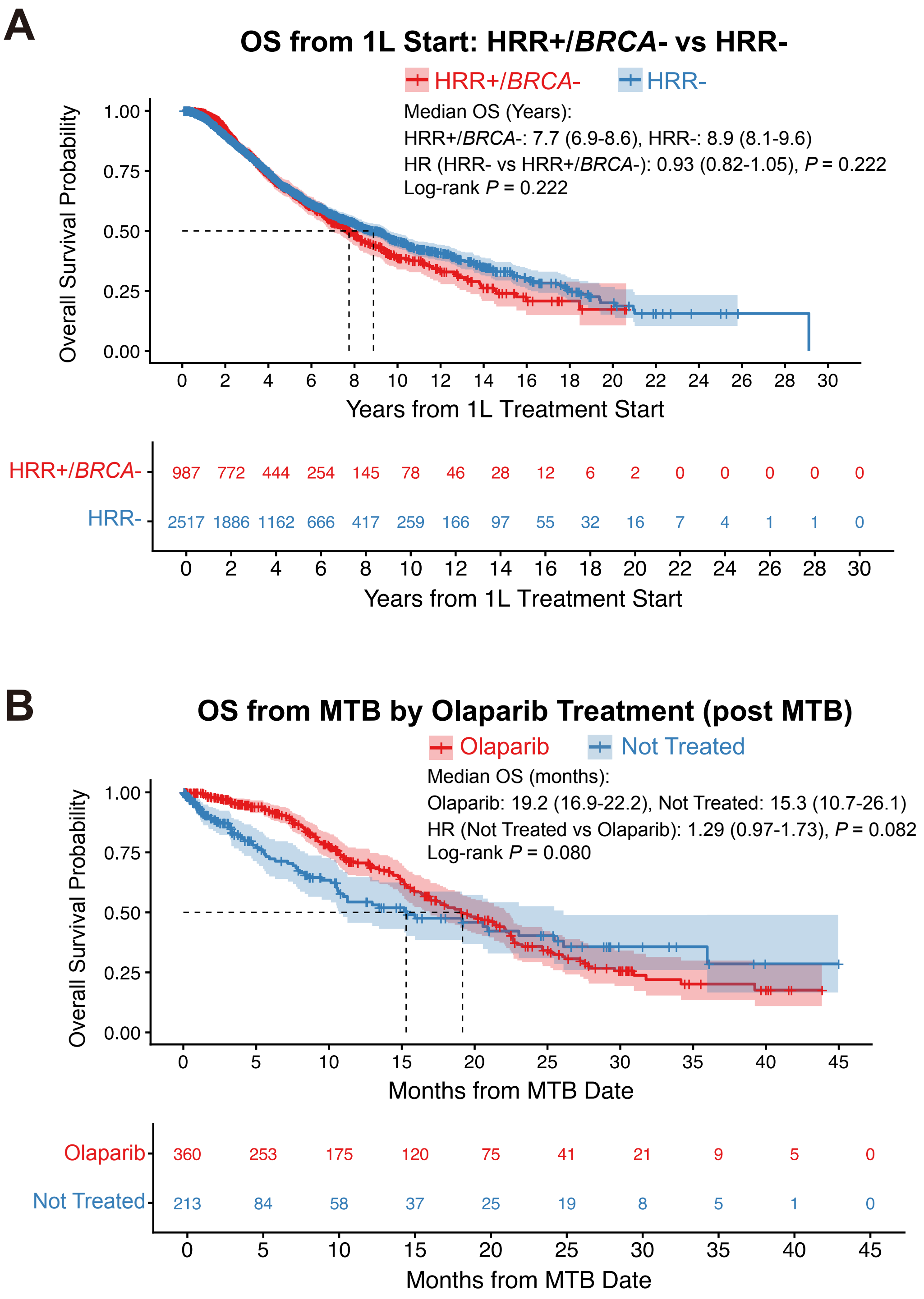


**Supplementary Figure 4.**

**Survival analyses in prostate cancer patients according to homologous recombination repair (HRR) gene status and olaparib treatment.**

**A)** Kaplan–Meier curves for overall survival (OS) from the initiation of first-line (1L) treatment comparing patients with HRR-positive but *BRCA*-negative tumors (HRR+/*BRCA*–) and those with HRR–wild-type tumors (HRR–).

**B)** Kaplan–Meier curves for OS from the date of molecular tumor board (MTB) discussion comparing patients with *BRCA1/2* pathogenic variants who received olaparib and those who were recommended olaparib by the MTB but ultimately did not receive olaparib.

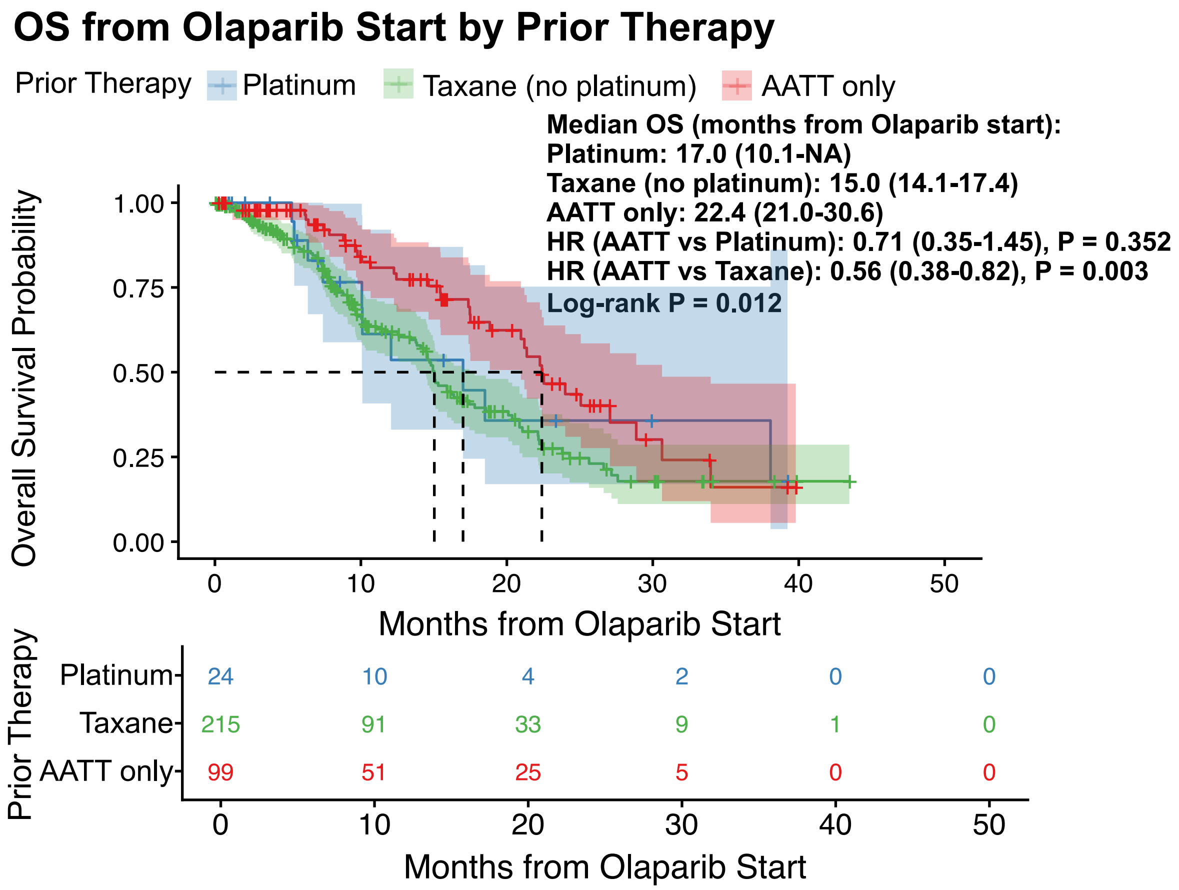


**Supplementary Figure 5.**

Survival analyses in prostate cancer patients harboring *BRCA* mutations according to prior therapy history following olaparib initiation.

Kaplan–Meier curves for overall survival following olaparib initiation (OS_olaparib), comparing patients previously treated with platinum-based therapy, taxane-based chemotherapy (without platinum), or androgen-axis-targeted therapy (AATT) alone.

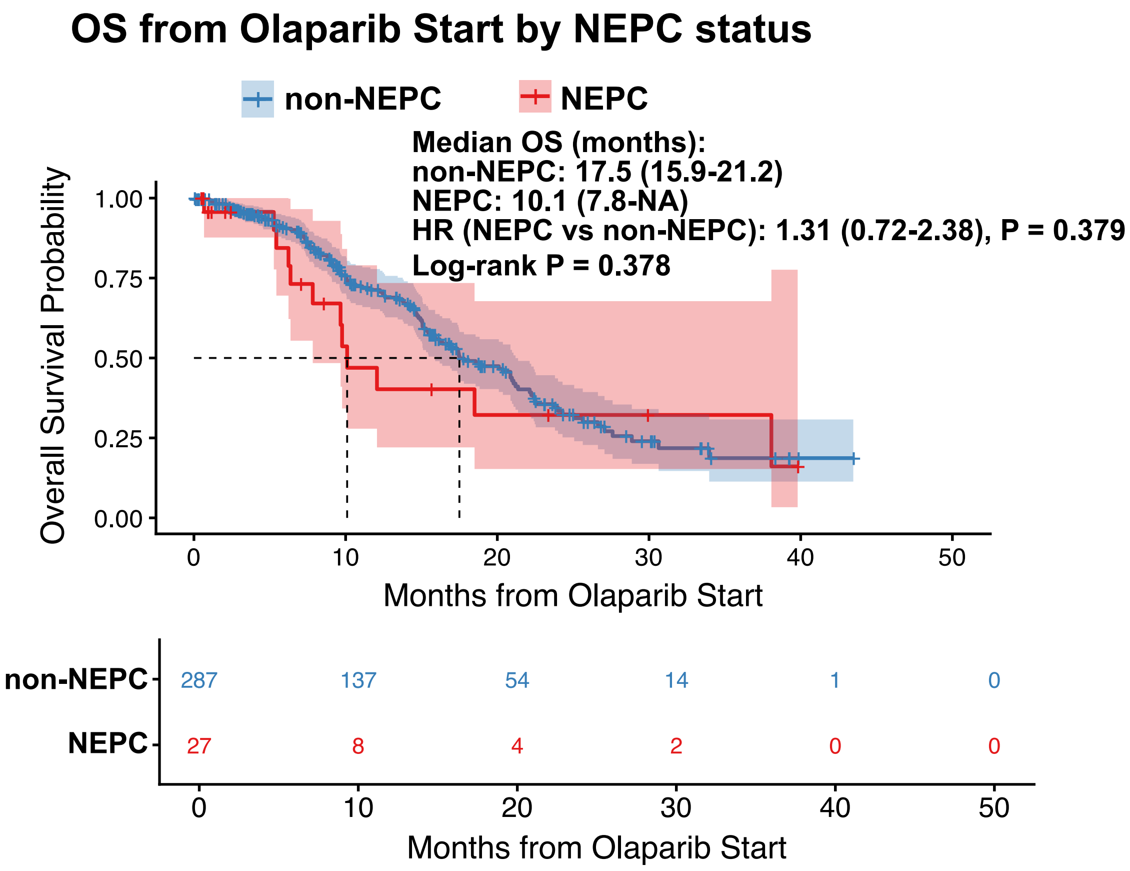


**Supplementary Figure 6.**

Survival analysis of prostate cancer patients harboring *BRCA2* mutations according to neuroendocrine prostate cancer (NEPC) status.

Kaplan–Meier curves of overall survival following olaparib initiation (OS_olaparib) comparing patients with and without a diagnosis of NEPC.

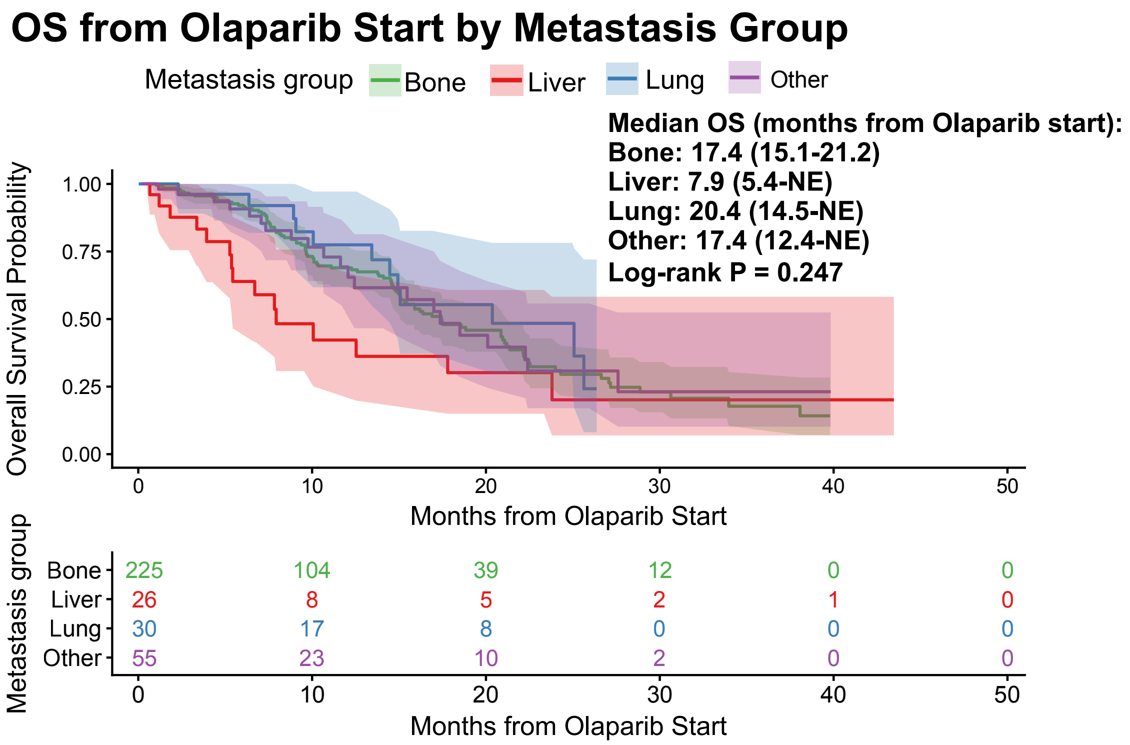


**Supplementary Figure 7.**

**Survival analyses following olaparib initiation in prostate cancer patients, stratified by metastatic site.**

Kaplan–Meier curves for overall survival following olaparib initiation (OS_olaparib) were generated for patients with liver metastases; lung metastases (excluding liver metastases); bone metastases (excluding liver and lung metastases); and other metastatic sites.

**Supplementary Table 1. Baseline characteristics of mCRPC patients stratified by *BRCA* pathogenic variants**

| Characteristics | Total n = 5,893 | *BRCA* PV n = 792 | *BRCA* WT n = 5,101 | p-value[b] *BRCA* PV vs *BRCA* WT |
|---|---|---|---|---|
| Age at Registration[a] | 73 (68–77) | 72 (66–77) | 73 (68–77) | **<0.01** |
| Age≥65 at Registration | | | | **<0.01** |
| <65 | 925 (16%) | 169 (21%) | 756 (15%) | |
| ≥65 | 4,968 (84%) | 623 (79%) | 4,345 (85%) | |
| PS at Registration | | | | 0.69 |
| 0 | 3,656 (62%) | 497 (63%) | 3,159 (62%) | |
| 1 | 1,810 (31%) | 244 (31%) | 1566 (31%) | |
| 2 | 188 (3%) | 25 (3%) | 163 (3%) | |
| 3 | 51 (1%) | 8 (1%) | 43 (1%) | |
| 4 | 4 (< 0.1%) | 0 (0%) | 4 (<0.1%) | |
| Unknown | 184 (3%) | 18 (2%) | 166 (3%) | |
| CGP testing | | | | **<0.01** |
| F1Liquid CDx | 2,210 (38%) | 250 (32%) | 1,960 (38%) | |
| FoundationOne CDx | 3,191 (54%) | 486 (61%) | 2,705 (53%) | |
| GenMineTOP | 49 (1%) | 8 (1%) | 41 (1%) | |
| Guardant360 CDx | 117 (2%) | 7 (1%) | 110 (2%) | |
| NCC OncoPanel | 326 (5%) | 41 (5%) | 285 (6%) | |
| MTB Timing | | | | 0.57 |
| 1L to 2L | 1,399 (24%) | 186 (23%) | 1,213 (24%) | |
| 3L to 4L | 1,883 (32%) | 263 (33%) | 1,620 (32%) | |
| 5L or later | 1,447 (25%) | 200 (25%) | 1,247 (24%) | |
| Unknown | 1,164 (20%) | 143 (18%) | 1,021 (20%) | |
| FH Breast | | | | **<0.01** |
| No | 5,216 (89%) | 641 (81%) | 4,575 (90%) | |
| Yes | 677 (11%) | 151 (19%) | 526 (10%) | |
| FH Ovarian | | | | **<0.01** |
| No | 5,821 (99%) | 766 (97%) | 5,055 (99%) | |
| Yes | 72 (1.2%) | 26 (3%) | 46 (1%) | |
| FH Prostate | | | | **<0.01** |
| No | 4,964 (84%) | 634 (80%) | 4,330 (85%) | |
| Yes | 929 (16%) | 158 (20%) | 771 (15%) | |
| FH Pancreas | | | | 0.88 |
| No | 5,503 (93%) | 741 (94%) | 4762 (93%) | |
| Yes | 390 (6.6%) | 51 (6%) | 339 (7%) | |
| Meta Bone | | | | 0.56 |
| No | 1,388 (24%) | 193 (24%) | 1,195 (23%) | |
| Yes | 4,505 (76%) | 599 (76%) | 3,906 (77%) | |
| Meta Lung | | | | 0.06 |
| No | 5,106 (87%) | 703 (89%) | 4,403 (86%) | |
| Yes | 787 (13%) | 89 (11%) | 698 (14%) | |
| Meta Liver | | | | 0.63 |
| No | 5,384 (91%) | 720 (91%) | 4,664 (91%) | |
| Yes | 509 (9%) | 72 (9%) | 437 (9%) | |
| Meta Lymph Node | | | | **<0.01** |
| No | 3,108 (53%) | 376 (47%) | 2,732 (54%) | |
| Yes | 2,785 (47%) | 416 (53%) | 2,369 (46%) | |
| Meta Brain | | | | 0.78 |
| No | 5,865 (99.5%) | 788 (99.5%) | 5,077 (99.5%) | |
| Yes | 28 (0.5%) | 4 (0.5%) | 24 (0.05%) | |
| Meta Other | | | | 0.58 |
| No | 5,392 (91%) | 729 (92%) | 4,663 (91%) | |
| Yes | 501 (9%) | 63 (8%) | 438 (9%) | |

[a]Median (Q1–Q3)
[b]P-values were calculated using Wilcoxon rank sum test, Fisher's exact test and Pearson's Chi-squared test. Bold indicates $p < 0.05$.
Abbreviations: PS, Performance Status; CGP, Cancer Genome Profiling; MTB, Molecular Tumor Board; FH, Family History

**Supplementary Table 2. Number of patients with pathogenic variants in HRR genes**

| HRR genes | Number of patients (n, ≥2) | % of patients (out of 2203) |
|---|---|---|
| *CDK12* | 686 | 31.14 |
| *BRCA2* | 624 | 28.33 |
| *ATM* | 379 | 17.20 |
| *CHEK2* | 78 | 3.54 |
| *PALB2* | 59 | 2.68 |
| *BRCA1* | 41 | 1.86 |
| *ATM, CHEK2* | 34 | 1.54 |
| *ATM, BRCA2* | 28 | 1.27 |
| *ATM, CDK12* | 26 | 1.18 |
| *CDK12, CHEK2* | 25 | 1.13 |
| *FANCA* | 20 | 0.91 |
| *BRIP1* | 18 | 0.82 |
| *BRCA2, CDK12* | 16 | 0.73 |
| *BARD1* | 15 | 0.68 |
| *BRCA2, CHEK2* | 15 | 0.68 |
| *RAD51D* | 13 | 0.59 |
| *BRCA2, PALB2* | 11 | 0.50 |
| *BRCA2, FANCA* | 8 | 0.36 |
| *FANCL* | 8 | 0.36 |
| *RAD51C* | 8 | 0.36 |
| *ATM, PALB2* | 7 | 0.32 |
| *BRCA1, BRCA2* | 6 | 0.27 |
| *CDK12, FANCA* | 5 | 0.23 |
| *CDK12, PALB2* | 5 | 0.23 |
| *BRCA1, CDK12* | 4 | 0.18 |
| *ATM, BARD1* | 3 | 0.14 |
| *ATM, BRCA2, CHEK2* | 3 | 0.14 |
| *ATM, BRIP1* | 3 | 0.14 |
| *BARD1, BRCA2* | 3 | 0.14 |
| *BRCA2, BRIP1* | 3 | 0.14 |
| *BRCA2, RAD51D* | 3 | 0.14 |
| *CHEK2, PALB2* | 3 | 0.14 |
| *ATM, BRCA1* | 2 | 0.09 |
| *ATM, BRCA1, BRCA2, CHEK2* | 2 | 0.09 |
| *ATM, BRCA2, CDK12, CHEK2* | 2 | 0.09 |
| *ATM, CDK12, CHEK2* | 2 | 0.09 |
| *BRCA1, CHEK2* | 2 | 0.09 |
| *BRCA2, CHEK2, RAD51D* | 2 | 0.09 |
| *BRCA2, FANCL* | 2 | 0.09 |
| *BRCA2, RAD51C* | 2 | 0.09 |
| *CDK12, RAD51C* | 2 | 0.09 |

**Supplementary Table 3. Types and frequencies of pathogenic *BRCA2* variants**

| *BRCA2* (n=830) | Number of variants (n) |
|---|---|
| loss | 162 |
| R2318* | 58 |
| I1859fs*3 | 45 |
| truncation | 24 |
| I605fs*9 | 15 |
| T3033fs*11 | 12 |
| W1692fs*3 | 12 |
| I605fs*11 | 10 |
| S1882* | 9 |
| D427fs*3 | 8 |
| I2675V | 8 |
| S2835* | 8 |
| P3039P | 7 |
| Q3026* | 6 |
| C1200fs*1 | 5 |
| K1691fs*15 | 5 |
| K467* | 5 |
| N1784fs*7 | 5 |
| N2135fs*3 | 5 |
| T3033fs*29 | 5 |
| E2877* | 4 |
| K1828fs*4 | 4 |
| K437fs*22 | 4 |
| L1908fs*2 | 4 |
| N1784fs*3 | 4 |
| N863fs*18 | 4 |
| R2520* | 4 |
| T3085fs*26 | 4 |
| T630fs*6 | 4 |
| V1447fs*1 | 4 |
| A2185fs*6 | 3 |
| A938fs*21 | 3 |
| K178fs*7 | 3 |
| K2217* | 3 |
| N986fs*2 | 3 |
| Q1925fs*2 | 3 |
| Q861* | 3 |
| A2864fs*5 | 2 |
| A338fs*11 | 2 |
| C2117fs*2 | 2 |
| E2558fs*7 | 2 |
| E2981fs*7 | 2 |
| F376fs*23 | 2 |
| G2748D | 2 |
| I2149fs*1 | 2 |

| | |
|---|---|
| K2657* | 2 |
| K3416fs*11 | 2 |
| L1904fs*5 | 2 |
| M2393fs*19 | 2 |
| N1287fs*2 | 2 |
| N257fs*17 | 2 |
| N319fs*8 | 2 |
| Q2163* | 2 |
| Q699fs*31 | 2 |
| R2784W | 2 |
| R2842C | 2 |
| R3052W | 2 |
| R3128* | 2 |
| S2219* | 2 |
| T2125fs*12 | 2 |
| T2125fs*4 | 2 |
| T269fs*7 | 2 |
| T3085fs*19 | 2 |
| V1532fs*2 | 2 |
| V464fs*3 | 2 |
| Y1710fs*1 | 2 |
| c.7806-2A>G | 2 |
| A1043fs*2 | 1 |
| A1237fs*2 | 1 |
| A1393V | 1 |
| A1395fs*15 | 1 |
| A1572fs*6 | 1 |
| A1648_V2280del | 1 |
| A1648fs*16 | 1 |
| A2857fs*6 | 1 |
| A2864fs*27 | 1 |
| A518fs*9 | 1 |
| A902fs*2 | 1 |
| C1893fs*6 | 1 |
| C822fs*3 | 1 |
| D1096fs*3 | 1 |
| D1575fs*3 | 1 |
| D1737fs*36 | 1 |
| D1807fs*8 | 1 |
| D2005fs*34 | 1 |
| D23fs*7 | 1 |
| D252fs*2 | 1 |
| D252fs*24 | 1 |
| D3064fs*11 | 1 |
| D479fs*6 | 1 |
| E1120* | 1 |
| E13* | 1 |

| | |
|---|---|
| E1308* | 1 |
| E1422* | 1 |
| E1441* | 1 |
| E1481fs*4 | 1 |
| E1550* | 1 |
| E1608* | 1 |
| E1608fs*6 | 1 |
| E1688fs*14 | 1 |
| E1755fs*6 | 1 |
| E1879* | 1 |
| E2029* | 1 |
| E2082* | 1 |
| E2089* | 1 |
| E2121* | 1 |
| E2198fs*4 | 1 |
| E2558* | 1 |
| E2598fs*50 | 1 |
| E2850fs*12 | 1 |
| E2923fs*1 | 1 |
| E2947fs*29 | 1 |
| E2981fs*37 | 1 |
| E394* | 1 |
| E462fs*6 | 1 |
| E612fs*25 | 1 |
| E714fs*5 | 1 |
| E790fs*20 | 1 |
| E804* | 1 |
| E808* | 1 |
| E826* | 1 |
| F1449fs*1 | 1 |
| F1870fs*4 | 1 |
| F2321fs*7 | 1 |
| F3139fs*24 | 1 |
| F333fs*16 | 1 |
| F625fs*19 | 1 |
| F81fs*19 | 1 |
| G106V | 1 |
| G1500* | 1 |
| G1500fs*3 | 1 |
| G1771fs*3 | 1 |
| G1892fs*17 | 1 |
| G2195fs*10 | 1 |
| G2379* | 1 |
| G2508fs*15 | 1 |
| G2529fs*22 | 1 |
| G3153fs*10 | 1 |
| H114fs*1 | 1 |

| | |
|---|---|
| H1223fs*9 | 1 |
| H1916fs*8 | 1 |
| H1932fs*12 | 1 |
| H477fs*7 | 1 |
| H543fs*14 | 1 |
| H543fs*17 | 1 |
| I1446fs*2 | 1 |
| I1490fs*24 | 1 |
| I1874fs*34 | 1 |
| I1945fs*18 | 1 |
| I2330fs*35 | 1 |
| I2675fs*6 | 1 |
| I332fs*3 | 1 |
| I383fs*12 | 1 |
| I383fs*16 | 1 |
| I729fs*21 | 1 |
| I79fs*17 | 1 |
| I80fs*18 | 1 |
| I860fs*6 | 1 |
| K1025* | 1 |
| K1492* | 1 |
| K1530fs*4 | 1 |
| K1565fs*3 | 1 |
| K1789fs*18 | 1 |
| K1860* | 1 |
| K1872fs*2 | 1 |
| K1881fs*27 | 1 |
| K1881fs*7 | 1 |
| K1959* | 1 |
| K21* | 1 |
| K2111* | 1 |
| K2150fs*19 | 1 |
| K2162fs*5 | 1 |
| K21_A22insATRCNK | 1 |
| K2308* | 1 |
| K2640fs*8 | 1 |
| K2657fs*15 | 1 |
| K314* | 1 |
| K3263fs*12 | 1 |
| K3326* | 1 |
| K3327fs*13 | 1 |
| K343fs*6 | 1 |
| K610fs*4 | 1 |
| K68fs*3 | 1 |
| K692fs*38 | 1 |
| K757* | 1 |
| K82fs*18 | 1 |

| | |
|---|---|
| K838fs*3 | 1 |
| K848fs*10 | 1 |
| K944* | 1 |
| K981fs*6 | 1 |
| L1208fs*4 | 1 |
| L1294fs*1 | 1 |
| L1387* | 1 |
| L146fs*6 | 1 |
| L1740fs*1 | 1 |
| L1993* | 1 |
| L2073* | 1 |
| L24* | 1 |
| L2428* | 1 |
| L2523fs*15 | 1 |
| L3027* | 1 |
| L440fs*12 | 1 |
| L474fs*12 | 1 |
| L557fs*1 | 1 |
| L698fs*10 | 1 |
| L74fs*19 | 1 |
| L759fs*13 | 1 |
| L760fs*2 | 1 |
| L88fs*12 | 1 |
| L947fs*13 | 1 |
| L971* | 1 |
| M1029fs*7 | 1 |
| M2235_E2236delinsI* | 1 |
| N1055fs*4 | 1 |
| N1066fs*10 | 1 |
| N1287fs*1 | 1 |
| N1316fs*2 | 1 |
| N1657fs*13 | 1 |
| N2030fs*10 | 1 |
| N2259fs*2 | 1 |
| N2259fs*21 | 1 |
| N2346fs*20 | 1 |
| N243fs*2 | 1 |
| N2556fs*93 | 1 |
| N30fs*6 | 1 |
| N372H | 1 |
| N517fs*6 | 1 |
| N588fs*26 | 1 |
| N812fs*13 | 1 |
| N986fs*5 | 1 |
| P1841fs*29 | 1 |
| P2204fs*2 | 1 |
| P2589fs*4 | 1 |

| | |
|---|---|
| P2771fs*6 | 1 |
| P3229fs*25 | 1 |
| P704fs*26 | 1 |
| P78fs*23 | 1 |
| P94fs*2 | 1 |
| Q1095fs*9 | 1 |
| Q126* | 1 |
| Q1429fs*19 | 1 |
| Q147* | 1 |
| Q1658* | 1 |
| Q1925* | 1 |
| Q1994* | 1 |
| Q1998* | 1 |
| Q2184fs*12 | 1 |
| Q2345* | 1 |
| Q2485* | 1 |
| Q2506* | 1 |
| Q2941* | 1 |
| Q3066* | 1 |
| Q3126* | 1 |
| Q3299* | 1 |
| Q407* | 1 |
| Q569* | 1 |
| Q569fs*20 | 1 |
| Q66fs*21 | 1 |
| Q73fs*20 | 1 |
| Q84fs*11 | 1 |
| Q940fs*5 | 1 |
| Q969* | 1 |
| R113fs*8 | 1 |
| R2336H | 1 |
| R2494* | 1 |
| R2602T | 1 |
| R2842fs*21 | 1 |
| R645* | 1 |
| R67fs*28 | 1 |
| R8fs*5 | 1 |
| S1001* | 1 |
| S1064fs*12 | 1 |
| S1079fs*11 | 1 |
| S1079fs*8 | 1 |
| S1099fs*1 | 1 |
| S1203fs*16 | 1 |
| S1248fs*11 | 1 |
| S1271fs*5 | 1 |
| S1341fs*33 | 1 |
| S1424fs*24 | 1 |

| | |
|---|---|
| S1650fs*16 | 1 |
| S1680fs*8 | 1 |
| S1733fs*1 | 1 |
| S1741fs*34 | 1 |
| S1741fs*36 | 1 |
| S1760fs*17 | 1 |
| S1764fs*3 | 1 |
| S1943fs*5 | 1 |
| S1946fs*3 | 1 |
| S1951fs*11 | 1 |
| S2148fs*2 | 1 |
| S2201fs*5 | 1 |
| S2243fs*37 | 1 |
| S2371fs*21 | 1 |
| S2533fs*18 | 1 |
| S2546fs*4 | 1 |
| S2670* | 1 |
| S2691fs*3 | 1 |
| S2697fs*6 | 1 |
| S273fs*4 | 1 |
| S2806fs*3 | 1 |
| S3080fs*3 | 1 |
| S313fs*2 | 1 |
| S3144fs*19 | 1 |
| S353* | 1 |
| S648fs*12 | 1 |
| S70fs*27 | 1 |
| S76fs*14 | 1 |
| S973* | 1 |
| T1282fs*2 | 1 |
| T1317fs*2 | 1 |
| T1410fs*9 | 1 |
| T1785fs*2 | 1 |
| T302fs*3 | 1 |
| T703fs*4 | 1 |
| T77fs*16 | 1 |
| V1166fs*15 | 1 |
| V1283fs*2 | 1 |
| V1639fs*3 | 1 |
| V2151fs*17 | 1 |
| V2174fs*17 | 1 |
| V220fs*4 | 1 |
| V2466A | 1 |
| V572fs*9 | 1 |
| W1563* | 1 |
| W194* | 1 |
| W2990* | 1 |

| | |
|---|---|
| W3191* | 1 |
| Y1739fs*1 | 1 |
| Y2047* | 1 |
| Y2215fs*13 | 1 |
| Y2826fs*38 | 1 |
| Y42fs*2 | 1 |
| Y592* | 1 |
| Y600* | 1 |
| Y71fs*27 | 1 |
| Y748* | 1 |
| Y828H | 1 |
| Y839fs*42 | 1 |
| c.1909+2T>C | 1 |
| c.316+1G>T | 1 |
| c.316+2T>C | 1 |
| c.316+2T>G | 1 |
| c.475+1G>A | 1 |
| c.476-12_481del18 | 1 |
| c.4821_6841+213del2234 | 1 |
| c.4963_6841+119del1998 | 1 |
| c.5294_6841+201del1749 | 1 |
| c.5354_6841+201del1689 | 1 |
| c.5751_6841+206del1297 | 1 |
| c.6030_6841+234delinsACTGTTTTAAGGG | 1 |
| c.6111_6841+203del934 | 1 |
| c.7436-23_7484del72 | 1 |
| c.7804_7805+4delinsT | 1 |
| c.7977-56_8121del201 | 1 |
| c.8331+2_8331+3delTA | 1 |
| c.8754+1G>C | 1 |
| c.8755-28_8794del68 | 1 |
| c.8954-88_9023del158 | 1 |
| c.9117G>A | 1 |
| c.9118-2A>C | 1 |
| R2669* | 1 |

**Supplementary Table 4. Types and frequencies of pathogenic *BRCA1* variants**

| ***BRCA1*** **(n=65)** | **Number of variants (n)** |
|---|---|
| truncation | 8 |
| L63* | 5 |
| loss | 5 |
| K654fs*47 | 4 |
| Q934* | 3 |
| A410fs*7 | 1 |
| C1697fs*1 | 1 |
| C24Y | 1 |
| C944fs*2 | 1 |
| E1148fs*7 | 1 |
| E797fs*3 | 1 |
| F1036fs*12 | 1 |
| F1761S | 1 |
| G677fs*5 | 1 |
| H692fs*19 | 1 |
| K1183* | 1 |
| K653fs*47 | 1 |
| K906fs*94 | 1 |
| L1854P | 1 |
| L954fs*15 | 1 |
| M1663V | 1 |
| M1663fs*16 | 1 |
| N1235fs*6 | 1 |
| Q1135* | 1 |
| Q1396* | 1 |
| Q687* | 1 |
| Q905* | 1 |
| R1203* | 1 |
| R1751* | 1 |
| R691fs*2 | 1 |
| S1147fs*1 | 1 |
| S1715C | 1 |
| S663fs*38 | 1 |
| T1685I | 1 |
| T1773I | 1 |
| T195fs*36 | 1 |
| T737fs*16 | 1 |
| V1088fs*2 | 1 |
| V660fs*13 | 1 |
| V923fs*76 | 1 |
| Y1853C | 1 |
| Y655fs*18 | 1 |
| c.4986+1G>T | 1 |
| c.5152+1_5152+28del28 | 1 |
| c.5468-1G>A | 1 |

**Supplementary Table 5. Summary of cases harboring pathogenic variants in both *BRCA1* and *BRCA2***

| case | *BRCA1* | *BRCA2* |
|---|---|---|
| 1 | Y655fs*18 | N1784fs*7 |
| 2 | R1751* | M1029fs*7 |
| 3 | K654fs*47 | K1691fs*15 |
| 4 | c.5468-1G>A | E2981fs*7, K1691fs*15, K178fs*7, W3191* |
| 5 | Y1853C | K2217* |
| 6 | S1147fs*1 | G3153fs*10 |
| 7 | truncation | c.7977-56_8121del201 |
| 8 | K654fs*47 | A1393V |
| 9 | c.5152+1_5152+28del28 | W1563* |
| 10 | L1854P | N812fs*13 |
| 11 | K1183P | K1691fs*15 |
| 12 | T195fs*36 | S1741fs*36 |

**Supplementary Table 6.**
**Patient characteristics stratified by *BRCA* pathogenic variants (Olaparib cohort)**

| Characteristics | *BRCA2* n = 314 | *BRCA1* n = 20 | *BRCA1+2* n = 4 | p-value[b] |
|---|---|---|---|---|
| Age at Registration[a] | 73 (66–77) | 72 (62–79) | 71 (68–76) | 0.66 |
| Age≥65 at Registration | | | | 0.18 |
| <65 | 69 (22%) | 7 (35%) | 1 (25%) | |
| ≥65 | 245 (78%) | 13 (65%) | 3 (75%) | |
| PS | | | | 0.81 |
| 0 | 203 (65%) | 15 (75%) | 4 (100%) | |
| 1 | 100 (32%) | 5 (25%) | 0 (0%) | |
| 2≦ | 7 (2%) | 0 (0%) | 0 (0%) | |
| Unknown | 4 (1%) | 0 (0%) | 0 (0%) | |
| Metastasis at Registration | | | | 0.93 |
| Lymph Node Only | 37 (12%) | 2 (10%) | 1 (25%) | |
| Bone ± Lymph Node | 208 (66%) | 14 (70%) | 3 (75%) | |
| Lung | 28 (9%) | 2 (10%) | 0 (0%) | |
| Liver | 24 (8%) | 2 (10%) | 0 (0%) | |
| Other[c] | 17 (5%) | 0 (0%) | 0 (0%) | |
| CGP testing | | | | 0.07 |
| F1Liquid CDx | 94 (30%) | 9 (45%) | 3 (75%) | |
| FoundationOne CDx | 205 (65%) | 10 (50%) | 1 (25%) | |
| Guardant360 CDx | 1 (<1%) | 1 (5%) | 0 (0%) | |
| NCC OncoPanel | 14 (5%) | 0 (0%) | 0 (0%) | |
| MTB Timing | | | | 0.95 |
| 1L to 2L | 73 (23%) | 5 (25%) | 0 (0%) | |
| 3L to 4L | 124 (39%) | 7 (35%) | 2 (50%) | |
| 5L or later | 99 (32%) | 7 (35%) | 2 (50%) | |
| Unknown | 18 (6%) | 1 (5.0%) | 0 (0%) | |
| Pre-treatment | | | | 0.80 |
| Androgen-axis–targeted therapy only | 94 (30%) | 5 (25%) | 0 (0%) | |
| Chemotherapy-exposed[d] | 220 (70%) | 15 (75%) | 4 (100%) | |
| Olaparib Timing | | | | 0.70 |
| 2-3L | 76 (24%) | 5 (25%) | 0 (0%) | |
| 4L | 61 (19%) | 4 (20%) | 0 (0%) | |
| 5L or more | 165 (53%) | 10 (50%) | 4 (100%) | |
| Unknown | 12 (4%) | 1 (5.0%) | 0 (0%) | |
| FH Breast | | | | 0.55 |
| No | 260 (83%) | 18 (90%) | 2 (50%) | |
| Yes | 54 (17%) | 2 (10%) | 2 (50%) | |
| FH Ovarian | | | | 0.59 |
| No | 301 (96%) | 19 (95%) | 4 (100%) | |
| Yes | 13 (4%) | 1 (5%) | 0 (0%) | |
| FH Prostate | | | | 1.00 |
| No | 247 (79%) | 16 (80%) | 3 (75%) | |
| Yes | 67 (21%) | 4 (20%) | 1 (25%) | |
| FH Pancreas | | | | 0.62 |
| No | 294 (94%) | 20 (100%) | 4 (100%) | |
| Yes | 20 (6%) | 0 (0%) | 0 (0%) | |

[a]Median (Q1–Q3)
[b]P-values (Wilcoxon for continuous; Fisher's exact for categorical) compare BRCA2 vs BRCA1 only.
Bold indicates $p < 0.05$.
[c]Including brain metastases.
[d]Previously treated with taxane and/or platinum.
Abbreviations: PS, Performance Status; CGP, Cancer Genome Profiling; MTB, Molecular Tumor Board; FH, Family History

**Supplementary Table 7.**
**Patient characteristics stratified by *BRCA2* pathogenic variants (Olaparib cohort)**

| Characteristics | Others n = 203 | *BRCA2* loss n = 72 | *BRCA2* R2318* n = 25 | *BRCA2* I1859fs*3 n = 18 | p-value[b] |
|---|---|---|---|---|---|
| Age at Registration[a] | 73 (67–77) | 73 (67–76) | 66 (59–75) | 72 (67–76) | 0.17 |
| Age≥65 at Registration | | | | | 0.31 |
| <65 | 40 (20%) | 17 (24%) | 9 (36%) | 4 (22%) | |
| ≥65 | 163 (80%) | 55 (76%) | 16 (64%) | 14 (78%) | |
| PS | | | | | 0.92 |
| 0 | 135 (67%) | 47 (65%) | 15 (60%) | 10 (56%) | |
| 1 | 60 (30%) | 23 (32%) | 10 (40%) | 7 (39%) | |
| 2≦ | 5 (2%) | 1 (1%) | 0 (0%) | 1 (5%) | |
| Unknown | 3 (1%) | 1 (1%) | 0 (0%) | 0 (0%) | |
| Metastasis at Registration | | | | | 0.76 |
| Lymph Node Only | 26 (13%) | 8 (11%) | 3 (12%) | 1 (6%) | |
| Bone ± Lymph Node | 136 (67%) | 47 (65%) | 16 (64%) | 12 (67%) | |
| Lung | 17 (8%) | 8 (11%) | 2 (8%) | 1 (6%) | |
| Liver | 13 (6%) | 4 (6%) | 4 (16%) | 3 (16%) | |
| Other[c] | 11 (6%) | 5 (7%) | 0 (0%) | 1 (5%) | |
| CGP testing | | | | | **<0.01** |
| F1Liquid CDx | 78 (38%) | 9 (13%) | 4 (16%) | 6 (33%) | |
| FoundationOne CDx | 112 (55%) | 63 (88%) | 20 (80%) | 11 (61%) | |
| Guardant360 CDx | 1 (0.5%) | 0 (0%) | 0 (0%) | 0 (0%) | |
| NCC OncoPanel | 12 (5.9%) | 0 (0%) | 1 (4.0%) | 1 (5.6%) | |
| MTB Timing | | | | | 0.73 |
| 1L to 2L | 40 (20%) | 18 (25%) | 9 (36%) | 6 (33%) | |
| 3L to 4L | 85 (42%) | 27 (38%) | 8 (32%) | 6 (33%) | |
| 5L or later | 65 (32%) | 23 (32%) | 7 (28%) | 6 (33%) | |
| Unknown | 13 (6.4%) | 4 (5.6%) | 1 (4.0%) | 0 (0%) | |
| Pre-treatment | | | | | 0.05 |
| Androgen-axis–targeted therapy only | 59 (29%) | 27 (38%) | 7 (28%) | 1 (6%) | |
| Chemotherapy-exposed[d] | 144 (71%) | 45 (62%) | 18 (72%) | 17 (94%) | |
| Olaparib Timing | | | | | 0.54 |
| 2-3L | 43 (21%) | 18 (25%) | 9 (36%) | 6 (33%) | |
| 4L | 44 (22%) | 10 (14%) | 5 (20%) | 2 (11%) | |
| 5L or more | 108 (53%) | 40 (56%) | 11 (44%) | 10 (56%) | |
| Unknown | 8 (3.9%) | 4 (5.6%) | 0 (0%) | 0 (0%) | |
| FH Breast | | | | | 0.14 |
| No | 162 (80%) | 65 (90%) | 19 (76%) | 16 (89%) | |
| Yes | 41 (20%) | 7 (9.7%) | 6 (24%) | 2 (11%) | |
| FH Ovarian | | | | | **<0.01** |
| No | 197 (97%) | 71 (99%) | 22 (88%) | 15 (83%) | |
| Yes | 6 (3.0%) | 1 (1.4%) | 3 (12%) | 3 (17%) | |
| FH Prostate | | | | | 0.90 |
| No | 160 (79%) | 57 (79%) | 20 (80%) | 13 (72%) | |
| Yes | 43 (21%) | 15 (21%) | 5 (20%) | 5 (28%) | |
| FH Pancreas | | | | | 0.21 |
| No | 190 (94%) | 70 (97%) | 22 (88%) | 16 (89%) | |
| Yes | 13 (6.4%) | 2 (2.8%) | 3 (12%) | 2 (11%) | |
| Yes | 15 (7.4%) | 7 (9.7%) | 4 (16%) | 4 (22%) | |

[a]Median (Q1–Q3)
[b]P-values were calculated using Kruskal-Wallis rank sum test, Fisher's exact test and Pearson's Chi-squared test. Bold indicates p < 0.05.
[c]Including brain metastases.
[d]Previously treated with taxane and/or platinum.
Abbreviations: PS, Performance Status; CGP, Cancer Genome Profiling; MTB, Molecular Tumor Board; FH, Family History
